# Diabetes, hypertension, and cardiovascular disease development

**DOI:** 10.1186/s12967-021-03217-2

**Published:** 2022-01-03

**Authors:** Fu-Shun Yen, James Cheng-Chung Wei, Lu-Ting Chiu, Chih-Cheng Hsu, Chii-Min Hwu

**Affiliations:** 1 Dr. Yen’s Clinic, No. 15, Shanying Road, Gueishan District, Taoyuan, 33354 Taiwan; 2 Department of Allergy, Immunology & Rheumatology, Chung Shan Medical University Hospital, No. 110, Sec. 1, Jianguo N. Rd., South District, Taichung City, 40201 Taiwan; 3 Institute of Medicine, Chung Shan Medical University, No. 110, Sec. 1, Jianguo N. Rd., South District, Taichung City, 40201 Taiwan; 4 Graduate Institute of Integrated Medicine, China Medical University, No.91, Hsueh-Shih Road, Taichung, 40402 Taiwan; 5 Management Office for Health Data, China Medical University Hospital, 3F., No.373-2, Jianxing Road, Taichung, 40459 Taiwan; 6 College of Medicine, China Medical University, No. 110, Sec. 1, Jianguo N. Rd., South District, Taichung City, 40201 Taiwan; 7 Institute of Population Health Sciences, National Health Research Institutes, 35 Keyan Road, Zhunan, Miaoli County 35053 Taiwan; 8 Department of Health Services Administration, China Medical University, No.91, Hsueh-Shih Road, Taichung, 40402 Taiwan; 9 Department of Family Medicine, Min-Sheng General Hospital, 168 ChingKuo Road, Taoyuan, 33044 Taiwan; 10 Department of Medicine, National Yang-Ming Chiao Tung University School of Medicine, No.155, Sec.2, Linong Street, Taipei, 11221 Taiwan; 11 Section of Endocrinology and Metabolism, Department of Medicine, Taipei Veterans General Hospital, No.201, Sec. 2, Shipai Road, Beitou District, Taipei, 11217 Taiwan

**Keywords:** Diabetes mellitus, Hypertension, Coronary artery disease, Stroke, Heart failure

## Abstract

**Background:**

We aimed to compare cardiovascular risks among participants with T2DM with and without subsequent HTN and participants with HTN with and without subsequent T2DM.

**Methods:**

From January 1, 2000, to December 31, 2018, we identified 16,236 matched pairs of T2DM participants with and without HTN (T2DM cohorts), 53,509 pairs of HTN participants with and without T2DM (HTN cohorts), and 21,158 pairs of comorbid HTN and T2DM participants with T2DM history or HTN history (comorbid cohorts) from Taiwan’s National Health Insurance Research Database. Cox proportional-hazard models were used to calculate the risk of cardiovascular disease.

**Results:**

The mean follow-up time of this study was 6.75 years. Mean incident rates of coronary artery disease for T2DM cohorts, HTN cohorts, and comorbid cohorts were 16.80, 23.18, and 31.53 per 1000 person-years, respectively. The adjusted hazard ratios (HRs) (95% confidence intervals [95% CIs]) for incident coronary artery disease, stroke, and heart failure in T2DM participants with versus without HTN were 2.22 (2.07–2.37), 1.19 (1.16–1.23), and 0.92 (0.82–1.02), respectively; the adjusted HRs for HTN participants with versus without T2DM were 1.69 (1.55–1.84), 1.25 (1.21–1.30), and 0.98 (0.93–1.05), respectively; the adjusted HRs for comorbid T2DM and HTN participants with previous T2DM versus previous HTN were 2.78 (2.37–3.27), 1.20 (1.13–1.28), and 0.95 (0.88–1.03), respectively.

**Conclusions:**

This nationwide cohort study demonstrated that both T2DM with subsequent HTN and HTN with subsequent diabetes were associated with higher cardiovascular disease risks.

## Background

The 2019 Global Burden of Disease Study indicated that ischemic heart disease, stroke, and hypertensive heart disease ranked as the first, second, and twenty-second leading causes of death, respectively, in persons aged 50–74 years [1]. The global number of patients with ischemic heart disease, stroke, and hypertensive heart disease was approximately 197.2, 101.5, and 18.6 million, respectively, in 2019 [2], which led to 9.1, 6.6, and 1.2 million cases of premature mortality, 176.6, 125.4, and 15.0 million years of life lost, and 5.4, 17.7, and 1.5 million years of living with a disability [2]. Both diabetes mellitus (DM) and hypertension (HTN) are important risk factors for atherosclerosis and cardiovascular disease (CVD) development [3]. Reports show that persons with type 2 diabetes mellitus (T2DM) are at 2.3 times (approximately) higher risk of cardiovascular disease than nondiabetics [3]; about one-third to two-thirds of T2DM patients died from CVD [3, 4]. Compared with non-HTN, HTN also significantly increased the risk and severity of CVD [5], and comorbid DM and HTN significantly increased cardiovascular risks [6–8].

Studies have reported that the treatment of hyperglycemia may reduce the risk of cardiovascular events [4, 9]. Numerous studies have reported that HTN treatment can reduce the risk of coronary artery disease, stroke, and heart failure [4, 9, 10]. A meta-analysis of randomized studies of comorbid persons with coexisting T2DM and HTN substantiated that a reduction in hemoglobin A1C (HbA1c) by 0.9% may reduce cardiovascular events by 9% [11], and systolic blood pressure reduction by 10 mmHg can reduce myocardial infarction by 12% and stroke events by 23% [12]. Another meta-analysis estimated that for every 200 persons with T2DM treated for 5 years, 3 myocardial infarction events could be prevented by a 0.9% reduction in HbA1c, and 14 events could be prevented by a 4 mmHg reduction in systolic blood pressure [13]. Diabetes, HTN, and lowering blood pressure or glucose may have different impacts on CVD risks. As there are few studies to evaluate the varying effects of diabetes and HTN on the risks of CVD, we conducted this study to determine if any difference exists in the risk of CVD in T2DM patients with or without subsequent HTN, hypertensive patients with or without subsequent T2DM, and patients with comorbid T2DM and HTN with previous T2DM or HTN history.

## Methods

### Data sources

The Taiwanese government has implemented National Health Insurance (NHI), a compulsory insurance system, since 1995; the government and employers pay most of the premiums, and people need to pay a small part only. By 2000, approximately 99% of the 29 million people in Taiwan were insured under this insurance program [14]. Taiwan's Ministry of Health and Welfare established the Health and Welfare Data Center (HWDC) in 2016 to control the use of national health insurance big data and standardize data management for all available healthcare data. The National Health Insurance Research Database (NHIRD) contains all medical records from 1995 to the present, including the data on the patient’s age, date of birth, sex, place of residence, therapy, and disease diagnosis according to the International Classification of Diseases, Ninth Revision, Clinical Modification (ICD-9-CM) and ICD-10-CM codes. This study consecutively recruited patients with newly diagnosed T2DM or HTN from the NHIRD between January 1, 2000, and December 31, 2018. Our study was approved by the Research Ethics Committee of China Medical University and Hospital (Approval No. CMUH109-109-REC2-031). All information of participants or care providers was encrypted before release to protect individual privacy; the study received a waiver of informed consent requirement from patients.

### Study design and definition of the study population

**Fig. 1:**
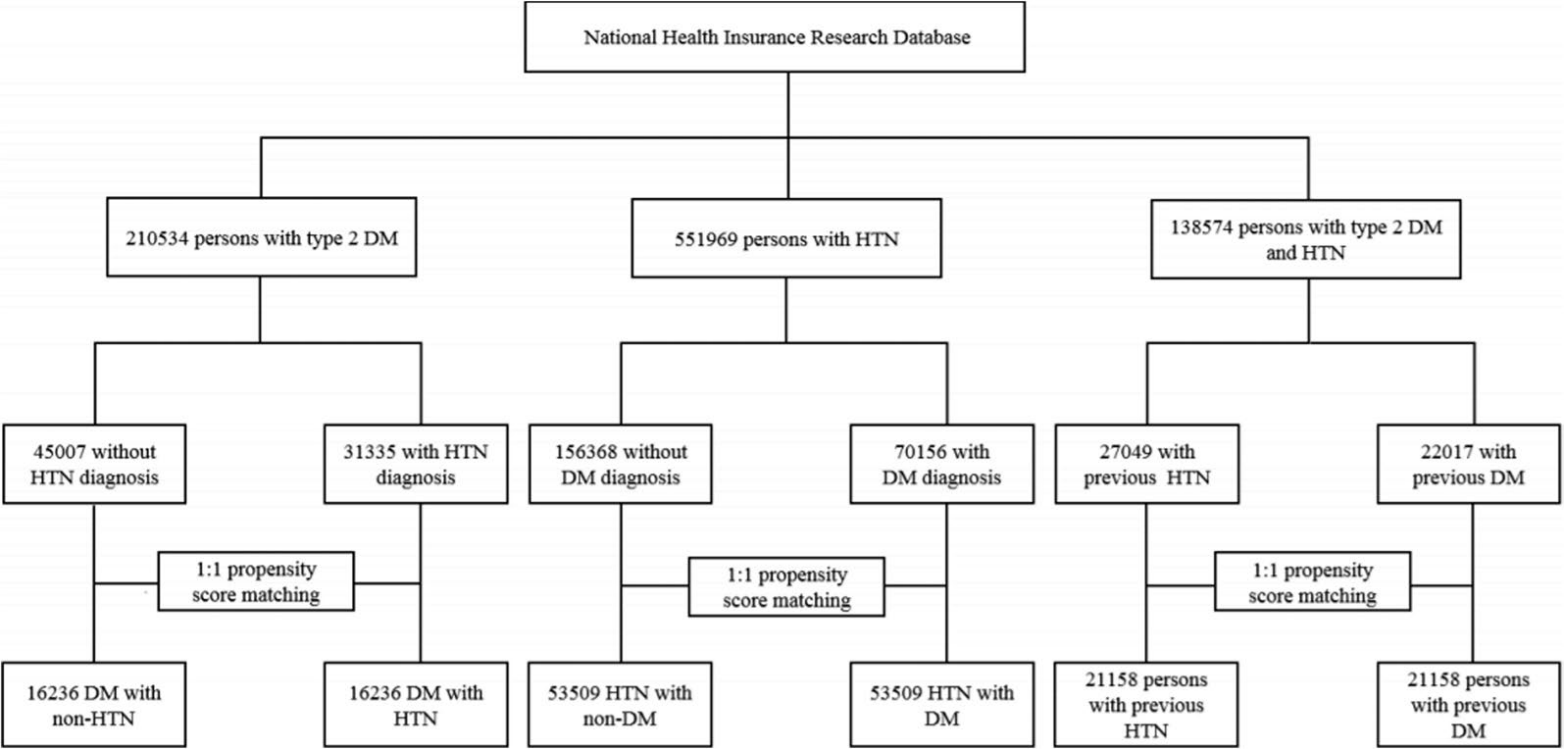
Flowchart of study population selection

In this nationwide retrospective population-based cohort study, we constructed 3 cohort studies (Fig. 1) from 2000 to 2017 to compare the risks of CVD for T2DM and HTN: (a) T2DM participants with and without HTN (T2DM cohorts) (b) HTN participants with and without T2DM (HTN cohorts) (c) participants with comorbid T2DM and HTN (comorbid cohorts). To define T2DM and HTN participants, T2DM was diagnosed based on ICD-9-CM code 250.xx or ICD-10-CM code E11, and HTN was diagnosed based on ICD-9-CM codes 401–405 and A26 or ICD-10-CM codes I10, I11, I12, I13, I15, and N26, with at least 2 outpatient claims within 1 year or one hospitalization. This method of defining T2DM and HTN diagnoses using ICD codes has been validated in previous studies [15, 16].

We divided the 2 10 534 participants with newly diagnosed T2DM (T2DM cohorts) into two groups based on subsequent HTN diagnoses. The index date was set as the date of HTN diagnosis for cases randomly assigned to controls. Participants with HTN history before T2DM were excluded. We divided the 5 51 969 participants with newly diagnosed HTN (HTN cohorts) into two groups based on subsequent T2DM diagnosis. The index date was set as the date of T2DM diagnosis for cases randomly assigned to controls. Participants with T2DM history before HTN were excluded. We categorized the 138,574 participants with comorbid T2DM and HT (comorbid cohorts) into two groups: (a) T2DM participants with HTN history, (b) HTN participants with T2DM history. The index date was set as the date of the last diagnosis for T2DM or HTN.

Exclusion criteria in this study were as follows: age below 20 years or above 80 years; unavailability of data on age or sex; the presence of type 1 DM (ICD-9-CM code 250.1x; ICD-10-CM E10), coronary artery disease, stroke, heart failure, atrial fibrillation (ICD-9-CM code 427; ICD-10-CM codes I45.0, I45.1, I45.2, I45.3, I45.4, I45.5, and I45.6), dialysis (ICD-9 codes V56.0, V56.8, and V45.1; ICD-10 codes Z49.31, Z49.32, and Z99.2), hepatic failure (ICD-9-CM codes 570, 572.2, 572.4, and 572.8; ICD-10-CM codes K72.00, K72.01, K72.10, K72.11, K72.90, K76.2, K72.90, K72.91, K76.7, and K76.81); year of index date before 2000. We also excluded participants who died or who were followed up < 180 days after the index date.

After the exclusion of ineligible participants, there were 31 335 patients with subsequent HTN and 45 007 participants without subsequent HTN in the T2DM cohorts. There were 70 156 participants with subsequent T2DM and 1 56 368 participants without subsequent T2DM in the HTN cohorts. The comorbid HTN and T2DM cohorts included 22 017 participants with previous T2DM and 27 049 participants with previous HTN.

### Comorbidities

The variables considered as potential confounders in this study were as follows: age, sex, and overweight (ICD-9-CM codes 278.02, 783.1, and V85.2; ICD-10-CM code R63.5); obesity (ICD-9-CM codes 278.00, 649.1, V77.8, and V85.3; ICD-10-CM codes E66.09, E66.1, E66.8, E66.9, and Z13.89); severe obesity (ICD-9-CM codes 278.01, 649.2, V45.86, and V85.4; ICD-10-CM codes E66.01 and E66.2); smoking status (ICD-9-CM codes 305.1, 649.0, and V15.82; ICD-10-CM codes F17.200, F17.201, F17.210, F17.220, F17.221, F17.290, F17.291, and Z87.891); dyslipidemia (ICD-9-CM code 272; ICD-10-CM codes E71.30, E71.31, E71.32, E71.39, E75.21, E75.22, E75.23, E75.24, E75.25, E75.29, E75.3, E75.4, E75.5, E75.6, E77, E78.0, E78.1, E78.2, E78.3, E78.4, E78.5, E78.6, E78.70, E78.71, E78.72, E78.79, E78.8, and E78.9); chronic kidney disease (CKD; ICD-9-CM codes 403.01, 403.11, 403.91, 404.02, 404.03, 404.12, 404.13, 404.92, 404.93, V42.0, and 790; ICD-10-CM codes E10.2, E10.65, E11.2, E11.65, E13.2, I12, I13, N03, N08, E10.21, E11.21, N05, N06, N07, N14, N15.0, N15.8, N15.9, N16, N17.1, N17.2, N19, and Z94.0); chronic obstructive pulmonary disease (COPD; ICD-9-CM codes 491, 492, and 496; ICD-10-CM codes J41, J42, J44, J43, and J44.9); liver cirrhosis (ICD-9-CM codes 571.5, 571.2, and 571.6; ICD-10-CM codes K70.2, K70.30, K70.31, K74.0, K74.1, K74.2, K74.60, K74.69, K74.3, K74.4, and K74.5); peripheral arterial occlusion disease (PAOD; ICD-9-CM codes 440.0, 440.20, 440.21, 440.22, 440.23, 440.24, 440.3, 440.4, 443.9, 443.81, and 443.89; ICD-10-CM codes I70.2, I70.92, I75.0, and I73.9); Charlson Comorbidity index (CCI) [17] and Diabetes Complication Severity Index (DCSI) scores [18]. Medication considerations were as follows: antidiabetic drugs, number of oral antidiabetic drugs, and insulin (Table 1); antihypertensive drugs and number of antihypertensive drugs (Table 2); statin; aspirin (Table 3). We also assessed DM duration (Table 1) and HTN duration (Table 2).

**Table 1:** Comparison of baseline characteristics in participants with type 2 DM

Characteristics	DM participants				SMD
	Non-HTN (n = 16,236)		HTN (n = 16,236)		
	N	%	n	%	
Age, years					
20–40	2087	12.85	2000	12.32	0.01
40–60	9803	60.38	9918	61.09	0.01
60–80	4346	26.77	4318	26.60	0.003
Mean ± SD	53.25 ± 11.92		53.32 ± 11.82		0.006
Gender					
Female	7507	46.24	7440	45.82	0.008
Male	8729	53.76	8796	54.18	0.008
Obesity					
Overweight	43	0.26	38	0.23	0.006
Obesity	270	1.66	239	1.47	0.01
Severe obesity	61	0.38	56	0.34	0.005
Smoking	343	2.11	311	1.92	0.01
Comorbidity					
Dyslipidemia	8796	54.18	9078	55.91	0.03
CKD	634	3.9	681	4.19	0.01
COPD	2975	18.32	3049	18.78	0.01
Liver cirrhosis	486	2.99	502	3.09	0.005
PAOD	333	2.05	350	2.16	0.007
CCI score					
0	6383	39.31	6099	37.56	0.03
1	4972	30.62	5132	31.61	0.02
≥ 2	4881	30.06	5005	30.83	0.01
DCSI score					
0	7397	45.56	7294	44.92	0.01
1	3057	18.83	3141	19.35	0.01
≥ 2	5782	35.61	5801	35.73	0.002
Antidiabetic drugs					
Metformin	5874	36.18	5967	36.75	0.01
Sulphonylurea	5391	33.20	5596	34.47	0.05
Thiazolidinedione	1197	7.37	1245	7.67	0.01
DPP-4 inhibitors	624	3.84	602	3.71	0.007
α-glucosidase inhibitor	1166	7.18	1234	7.60	0.012
Number of antidiabetic drugs					
0–1	11,686	71.98	11,569	71.26	0.01
2–3	4180	25.75	4295	26.45	0.011
≥ 3	370	2.28	372	2.29	0.008
Insulin	4561	28.09	4718	29.06	0.02
Other drug					
Statin	3673	22.62	3762	23.17	0.01
DM duration, year	3.72 ± 3.26		3.78 ± 3.84		0.01

Data are shown as n (%) or mean ± SD. *DM* diabetes mellitus, *HTN* hypertension, *CKD* chronic kidney disease, *COPD* chronic obstructive pulmonary disease, *PAOD* peripheral arterial occlusion disease, *CCI* Charlson comorbidity index, *DCSI* diabetes complications severity index, *SMD* standardized mean difference. A standardized mean difference of 0.05 or less indicates a negligible difference

**Table 2:** Comparison of baseline characteristics in participants with HTN

Characteristics	HTN participants				SMD
	Non-DM (n = 53,509)		DM (n = 53,509)		
	N	%	n	%	
Age, years					
20–40	4923	9.20	4555	8.51	0.03
40–60	28,831	53.88	30,124	56.30	0.04
60–80	19,755	36.92	18,830	35.19	0.02
Mean ± SD	56.48 ± 12.81		56.20 ± 11.71		0.01
Gender					
Female	25,549	47.75	25,531	47.71	0.001
Male	27,960	52.25	27,978	52.29	0.001
Obesity					
Overweight	75	0.14	81	0.15	0.002
Obesity	695	1.30	743	1.39	0.007
Severe obesity	152	0.28	146	0.27	0.002
Smoking	824	1.54	856	1.60	0.005
Comorbidity					
Dyslipidemia	24,512	45.80	24,907	46.55	0.01
CKD	2187	4.09	2318	4.33	0.01
COPD	10,096	18.87	10,785	20.16	0.03
Liver cirrhosis	826	1.54	889	1.66	0.01
PAOD	1079	2.02	1093	2.04	0.001
CCI score					
0	22,517	42.08	21,167	39.56	0.05
1	16,583	30.99	17,398	32.51	0.03
≥ 2	14,409	26.93	14,944	27.93	0.02
Antihypertensive drugs					
ACEI/ARB	26,416	49.37	26,930	50.33	0.01
β-blockers	25,418	47.50	26,475	49.48	0.03
Calcium-channel blockers	28,200	52.70	28,960	54.12	0.02
Diuretics	19,473	36.39	20,124	37.61	0.02
Number of antihypertensive drugs					
0–1	22,709	42.44	21,851	40.84	0.03
2–3	24,681	46.12	24,241	45.30	0.01
≥ 3	6119	11.44	7417	13.86	0.04
Other drug					
Statin	9976	18.64	10,523	19.67	0.02
Aspirin	9721	18.17	10,069	18.82	0.01
HTN duration, year	3.81 ± 3.29		3.91 ± 3.86		0.02

Data are shown as n (%) or mean ± SD. *DM* diabetes, *HTN* hypertension, *CKD* chronic kidney disease, *COPD* chronic obstructive pulmonary disease, *PAOD* peripheral arterial occlusion disease, *ACEI* angiotensin-converting enzyme inhibitor, *ARB* angiotensin receptor blocker, *CCI* Charlson comorbidity index, *SMD* standardized mean difference. A standardized mean difference of 0.05 or less indicates a negligible difference

**Table 3:** Comparison of baseline characteristics in participants with coexistence of DM and HTN

Characteristics	DM and HTN participants				SMD
	With previous HTN (n = 21,158)		With previous DM (n = 21,158)		
	N	%	N	%	
Age, years					
20–40	1368	6.47	1483	7.01	0.02
40–60	11,131	52.61	10,945	51.73	0.01
60–80	8659	40.93	8730	41.26	0.006
Mean ± SD	57.61 ± 11.85		57.50 ± 11.92		0.01
Gender					
Female	10,106	47.76	10,114	47.8	0.008
Male	11,052	52.24	11,044	52.2	0.008
Obesity					
Overweight	33	0.16	37	0.17	0.004
Obesity	189	0.89	214	1.01	0.01
Severe obesity	41	0.19	49	0.23	0.008
Smoking	243	1.15	266	1.26	0.01
Comorbidity					
Dyslipidemia	10,059	52.27	11,185	52.86	0.01
CKD	951	4.49	1035	4.89	0.01
COPD	4097	19.36	1079	19.28	0.00
Liver cirrhosis	520	2.45	793	3.74	0.01
PAOD	467	2.21	489	2.31	0.007
DCSI score					
0	10,277	48.57	7989	37.76	0.01
1	2376	11.23	3016	14.25	0.01
≥ 2	8505	40.20	10,153	47.99	0.02
CCI score					
0	10,433	49.31	9134	43.17	0.21
1	6055	28.62	6515	30.79	0.09
≥ 2	4670	22.07	5509	26.04	0.15
Antidiabetic drugs					
Metformin	3418	16.15	7344	34.71	0.44
Sulphonylurea	3068	14.50	7582	35.84	0.51
Thiazolidinedione	165	0.78	1601	7.57	0.34
DPP-4 inhibitors	123	0.58	548	2.59	0.16
α-glucosidase inhibitor	290	1.37	1471	6.95	0.28
Number of antidiabetic drugs					
0–1	19,716	93.18	15,170	71.70	0.58
2–3	1435	6.78	5543	26.2	0.54
≥ 3	7	0.03	445	2.10	0.2
Insulin	4440	20.98	5388	25.47	0.1
Antihypertensive drugs					
ACEI/ARB	9753	46.10	6384	30.17	0.33
β-blockers	9687	45.78	5509	26.04	0.42
Calcium-channel blockers	10,140	47.93	5757	27.21	0.44
Diuretics	7041	33.28	4137	19.55	0.43
Number of antihypertensive drugs					
0–1	9843	46.52	15,407	72.82	0.55
2–3	9122	43.11	5503	26.01	0.36
≥ 3	2193	10.36	248	1.17	0.40
Other drug					
Statin	4065	19.21	4090	19.33	0.003
Aspirin	2678	12.66	2836	13.40	0.02
HTN duration, year	3.45 ± 3.41		–		
DM duration, year	–		3.42 ± 3.24		

Data are shown as n (%) or mean ± SD. *DM* diabetes, *HTN* hypertension, *CKD* chronic kidney disease, *COPD* chronic obstructive pulmonary disease, *PAOD* peripheral arterial occlusion disease, *ACEI* angiotensin-converting enzyme inhibitor, *ARB* angiotensin receptor blocker, *CCI* Charlson comorbidity index, *DCSI* diabetes complications severity index, *SMD* standardized mean difference. A standardized mean difference of 0.05 or less indicates a negligible difference

### Main outcomes

We investigated the development of coronary artery disease (CAD; ICD-9-CM codes 410–414; ICD-10-CM codes I20, I21, I22, I24, I25.1, I25.2, I25.3, I25.4, I25.5, I25.6, I25.7, I25.81, I25.82, I25.83, I25.84, I25.89, and I25.9), stroke (ICD-9-CM codes 430–438; ICD-10-CM codes G45.0, G45.1, G45.2, G45.3, G45.4, G45.8, G45.9, G46, I60, I61, I62, I63, I65, I66, I67.0, I67.1, I67.2, I67.3, I67.4, I67.5, I67.6, I67.7, I67.8, I67.9, I68, and I69), and heart failure (ICD-9-CM codes 398.91, 402.01, 402.11, 402.91, 404.01, 404.03, 404.11, 404.13, 404.91, 404.93, 428, and 429.4; ICD-10-CM codes I09.81, I11.0, I13.0, I13.2, I50, I97.0, I97.110, I97.111, I97.120, I97.121, I97.130, I97.131, I97.190, and I97.191), with at least 2 outpatient claims within 1 year or one hospitalization from 2000 to 2018. This method of ICD codes to define cardiovascular events has been validated in previous studies in Taiwan [16, 19, 20]. The incidence rates of coronary artery disease, stroke, and heart failure were calculated and compared between the study and control participants. All study participants were followed until they were diagnosed with any outcomes, withdrew from the NHI system, or until December 31, 2018, whichever came first.

### Statistical analysis

We adopted propensity-score matching to optimize comparability between the study and control groups [21]. We estimated the propensity score for every participants through nonparsimonious multivariable logistic regression and used approximately 20 clinically related variables in the analysis as control variables (Tables 1, 2, 3). The nearest-neighbor algorithm was used to generate matching pairs assuming that the proportion of 0.995–1.0 is optimal.

In descriptive statistics, a chi-square test and Student’s t-test were used to evaluate the allocation of category and continuous variables, respectively. A standardized mean difference of less than 0.1 was considered balanced distribution between the cases and controls. Crude and multivariate-adjusted Cox proportional-hazard models with robust sandwich standard error estimates were used to compare the risk of outcomes between the study and control groups. The proportional hazards assumption was not violated by comparing estimated log–log survival curves for all time-independent covariates. The results are presented as hazard ratios (HRs) and 95% confidence intervals (CIs) for the study and control groups. The incidence rates of outcomes were measured by the number of cases per 1,000 person-years. Person-years was the time from the index date to the date of events, death, or the end of follow-up (December 31, 2018), whichever occurred first.

Two-tailed *P* < 0.05 was considered significant. SAS v9.4 (SAS Institute, Inc., Cary, NC, USA) was used for analyses.

## Results

### Baseline characteristics

Demographic and clinical characteristics of the three study cohorts are presented in Tables 1, 2, 3. After propensity score matching, the distributions of all characteristics were similar between the cases and controls in the three cohorts (SMD < 0.1). In the DM cohorts, after propensity matching by age, sex, obesity, smoking, comorbidity, DCSI score, antidiabetic drugs, statins, and diabetes duration, 16 236 pairs of matched participants were selected (Table 1). The mean follow-up time was 6.46 and 7.39 years for T2DM participants with subsequent HTN and without subsequent HTN, respectively. In the HTN cohorts, after propensity matching by age, sex, obesity, smoking, comorbidity, antihypertensive diabetic drugs, statins, and HTN duration, 53 509 pairs of matched participants were selected (Table 2). The mean follow-up time was 6.57 and 7.02 years for HTN participants with subsequent T2DM and without subsequent T2DM, respectively. In the comorbid T2DM and HTN cohorts, after propensity matching by age, sex, obesity, smoking, comorbidity, DCSI score, statins, and aspirin, 21 158 pairs of matched patients were selected (Table 3). The mean follow-up time was 6.43 and 6.61 years for comorbid T2DM and HTN participants with previous T2DM and previous HTN, respectively.

### Main outcomes

Table 4 demonstrates the overall incidence rates of coronary artery disease, stroke, and heart failure between the cases and controls among the three cohorts. For the risk of coronary artery disease development, in the T2DM cohorts, 2514 (15.48%) participants with subsequent HTN and 1353 (8.33%) without subsequent HTN developed coronary artery disease during the follow-up period (incidence rate of 22.68 vs. 10.91 per 1000 person-years). The multivariable-adjusted HR (95% CI) for participants with subsequent HTN compared to participants without HTN was 2.22 (2.07–2.37). In the HTN cohorts, 9690 (18.11%) participants with subsequent T2DM and 8540 (15.96%) without subsequent T2DM developed coronary artery disease during the follow-up period (incidence rate of 25.22 vs. 21.14 per 1000 person-years). The adjusted HR (95% CI) for patients with subsequent T2DM compared to participants without T2DM was 1.19 (1.16–1.23). In the comorbid DM and HTN cohorts, 4847 (22.91%) participants with previous T2DM and 4962 (23.45%) participants with previous HTN developed coronary artery disease during the follow-up period (incidence rate of 31.56 vs. 31.50 per 1000 person-years). The adjusted HR (95% CI) for patients with previous T2DM compared to participants with previous HTN was 0.92 (0.82–1.02).

**Table 4:** HRs and 95% CIs for the outcomes of cardiovascular diseases

Outcomes	DM participants						Crude HR (95% CI)	Adjusted HR^a^ (95% CI)
	Without HTN (n = 16,236)			With HTN (n = 16,236)				
	Events	PY	IR	Events	PY	IR		
CAD	1353	124,027	10.91	2514	110,871	22.68	2.21 (2.07–2.38)	2.22 (2.07–2.37)
Stroke	863	126,799	6.81	1349	119,087	11.33	1.64 (1.50–1.78)	1.69 (1.55–1.84)
Heart failure	208	130,144	1.60	564	123,184	4.58	2.82 (2.40–3.31)	2.78 (2.37–3.27)

Outcomes	HTN participants						Crude HR (95% CI)	Adjusted HR^b^ (95% CI)
	Without DM (n = 53,509)			With DM (n = 53,509)				
	Events	PY	IR	Events	PY	IR		
CAD	8540	403,982	21.14	9690	384,191	25.22	1.18 (1.15–1.22)	1.19 (1.16–1.23)
Stroke	5236	431,530	12.13	6081	415,229	14.64	1.20 (1.16–1.25)	1.25 (1.21–1.30)
Heart failure	1796	453,406	3.96	2040	442,778	4.61	1.16 (1.09–1.24)	1.20 (1.13–1.28)

Outcomes	Coexisted DM and HTN participants						Crude HR (95% CI)	Adjusted HR^c^ (95% CI)
	Previous HTN (n = 21,158)			Previous DM (n = 21,158)				
	Events	PY	IR	Events	PY	IR		
CAD	4962	157,525	31.50	4847	153,584	31.56	0.99 (0.85–1.03)	0.92 (0.82–1.02)
Stroke	3099	174,743	17.73	3374	167,244	20.17	1.12 (1.07–1.18)	0.98 (0.93–1.05)
Heart failure	1196	189,855	6.30	1319	181,499	7.27	1.16 (1.08–1.25)	0.95 (0.88–1.03)

*DM* diabetes mellitus, *HTN* hypertension, *CAD* coronary artery disease, *PY* person-years, *IR* incidence rate, per 1000 person-years, *HR* hazard ratio, *CI* confidence interval. ^a^ aHR adjusted for age, sex, obesity, smoking, dyslipidemia, chronic kidney disease (CKD), chronic obstructive pulmonary disease (COPD), liver cirrhosis, peripheral arterial occlusive disease (PAOD), Charlson comorbidity index (CCI) score, Diabetes Complications Severity Index (DCSI) score, antidiabetic drugs, statin, DM duration and index year. ^b^ aHR adjusted for age, gender, obesity, smoking, dyslipidemia, CKD, COPD, liver cirrhosis, PAOD, CCI score, antihypertensive drugs, statin, aspirin, HTN duration, and index year. ^c^ aHR adjusted for age, sex, obesity, smoking, dyslipidemia, CKD, COPD, liver cirrhosis, PAOD, statin, aspirin, and index year

For the risk of stroke, the adjusted HRs (95% CIs) for incident stroke in T2DM participants with HTN and without HTN, HTN participants with T2DM and without T2DM, and comorbid DM and HTN participants with previous T2DM and previous HTN were 1.69 (1.55–1.84), 1.25 (1.21–1.30), and 0.98 (0.93–1.05), respectively. For risk of heart failure, the adjusted HRs (95% CIs) for incident heart failure in T2DM participants with HTN and without HTN, HTN participants with T2DM and without T2DM, comorbid DM and HTN participants with previous T2DM and previous HTN were 2.78 (2.37–3.27), 1.20 (1.13–1.28), and 0.95 (0.88–1.03), respectively.

**Fig. 2:**
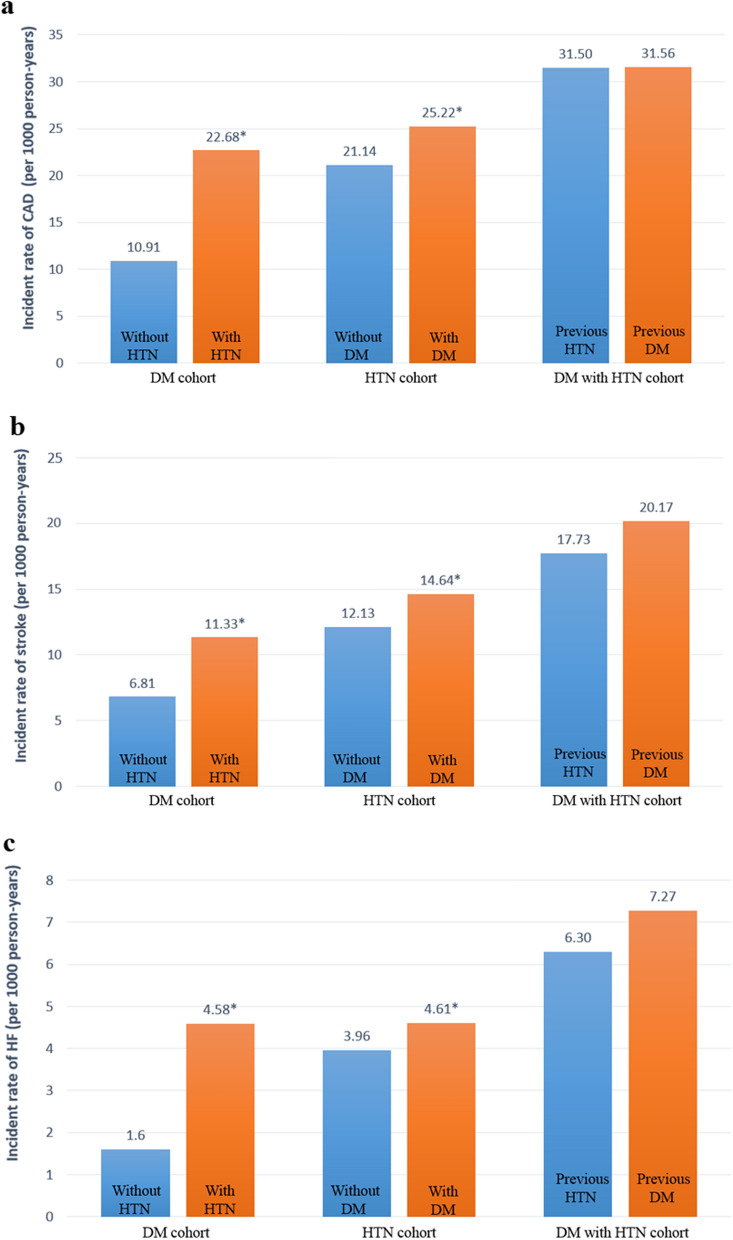
Comparison of the incidence rates of cardiovascular outcomes. **a** coronary artery disease (CAD); **b** stroke; **c** heart failure (HF). The numbers on the top of each bar graph indicate the incidence rates of cardiovascular disease. ^*^P < .0001 indicates significantly different aHRs of outcomes between study and comparison groups

In these 3 cohorts, the mean incident rates of coronary artery disease, stroke, and heart failure were 23.84, 13.80, and 4.72 per 1000 person-years, respectively (Table 4). Figure 2 separately presents the incidence rates of coronary artery disease, stroke, and heart failure among these cohorts. From Fig. 2, comorbid DM and HTN participants seemed to have a higher risk of CVD than HTN patients, and HTN participants had a higher risk of CVD than T2DM participants. T2DM participants with subsequent HTN compared to participants without HTN and HTN participants with subsequent T2DM compared to participants without T2DM had significantly higher risks of coronary artery disease, stroke, and heart failure (Table 4 and Fig. 2). The increased CVD risk for T2DM participants with HTN compared to participants without HTN was higher than that for HTN participants with T2DM compared to participants without T2DM (Fig. 2).

## Discussion

This large-series cohort study demonstrated the following: (1) In participants with T2DM or HTN, the incidence rate of coronary artery disease was higher than that of stroke and heart failure. (2) Comorbid DM and HTN participants had a higher risk of CVD than HTN participants, and HTN participants had a higher risk of CVD than T2DM participants. (3) T2DM with subsequent HTN was associated with a significantly higher risk of CVD than T2DM without HTN; HTN with subsequent T2DM was associated with a significantly higher risk of CVD than HTN without T2DM. (4) The increased CVD risk of T2DM with subsequent HTN was higher than that of HTN with subsequent T2DM.

In most countries, coronary artery disease is the main cardiovascular disease in patients with T2DM or HTN. However, in other countries, stroke is the main cardiovascular event [2]. The incidence of coronary artery disease was higher than that of stroke in this study. In the United States, due to advances in treatment, coronary artery disease and stroke incidence have significantly reduced, whereas heart failure incidence has gradually increased [22]. However, the incidence of stroke and coronary artery disease was still much higher than that of heart failure in this study. Taiwan’s 2019 Diabetes Atlas also revealed that patients with T2DM had higher incidence rates of coronary artery disease than those with stroke and heart failure [23].

Our study revealed that T2DM with subsequent HTN, HTN with subsequent T2DM, and comorbid T2DM and HTN were associated with higher risks of CVD. HTN may accelerate the deleterious effects of diabetes for CVD development, and diabetes may potentiate the detrimental effects of HTN for CVD development. This phenomenon has been reported by previous studies, in which individuals with combined HTN and T2DM had a higher risk of cardiovascular events [6, 7, 24, 25]. The interactive mechanisms may be responsible for the accelerated development of CVD in patients with diabetes and HTN. Our study demonstrated that HTN compared to T2DM cohorts, and T2DM patients with subsequent HTN compared to those without subsequent HTN had higher risks of CVD. HTN seemed to confer a higher risk of CVD than T2DM. Meta-analyses have also demonstrated that the reduction of systolic blood pressure has a greater impact on the decrease in CVD risk than the reduction of blood glucose [4, 11–13, 26]. To explain this observation, the possible mechanisms of CVD caused by diabetes and HTN need consideration.

Hyperglycemia in diabetes triggers the generation of reactive oxygen species (ROS) and advanced glycation end-products, leading to the initiation and progression of atherosclerosis. ROS can induce endothelial dysfunction [4, 8], trigger blood coagulability, and increase thrombosis risk in vessels. ROS can also lead to microvascular complications and cardiomyopathy, which may increase the risk of heart failure. Chronic hyperglycemia can produce low-grade systemic inflammation, cause thrombosis in atherosclerotic arteries, and promote the development of cardiovascular events. Hyperglycemia and insulin resistance can activate the renin–angiotensin–aldosterone system and increase the risk of CVD [4, 26]. HTN patients usually have metabolic syndrome caused by insulin resistance and hyperactivity of the sympathetic tone, which can activate the renin–angiotensin–aldosterone system and promote atherosclerosis development. Chronic HTN is associated with increased vascular inflammation, oxidative stress, endothelial dysfunction, hyperviscosity, and a prothrombotic state. Mechanical stress is the main factor for HTN that leads to the development of atherosclerosis and includes three-dimensional forces: shear stress, transmural pressure, and wall stress. Studies show that shear stress activates angiotensin II in HTN patients; transmural pressure produces a net pressure effect on endothelial cells and vascular smooth muscle cells. Wall stress can stretch smooth cells, increasing angiotensinogen-converting enzyme activity and cell growth, and finally cause muscle cell hypertrophy. Mechanical stress can cause irreversible elastin fragmentation and collagen deposition in blood vessels, leading to arterial stiffness [27]. Moreover, a long-term hypertensive state can lead to cardiac hypertrophy and increase the risks of myocardial infarction and heart failure [27, 28]. The specific factor of mechanical stress induced by HTN may have a greater impact on CVD occurrence.

People without HTN or patients with diabetes who have not developed HTN should avoid weight gain and obesity, avoid an unhealthy diet or excessive sodium intake, ensure adequate potassium intake, stay physically active, and reduce alcohol consumption [29]. These measures may reduce the occurrence of HTN, stabilize atherosclerotic status, and reduce cardiovascular events.

This study has some strengths. First, Taiwan’s NHI program is mandatory and covers approximately all people of this country (99%). This nationwide cohort study recruited patients from the NHIRD, which may reduce selection bias. Second, the data collection period was approximately 18 years from 2000 to 2018, sufficiently long for observing CVD occurrence. However, this study has some limitations. First, this study lacks data on blood pressure, glucose, HbA_1C_, biochemical results, CT images, and echocardiograms for HTN, T2DM, and CVD diagnosis. We used ICD codes for disease diagnosis; this method has been validated in previous studies with acceptable accuracy. The study lacked blood pressure and glucose data; therefore, we had no information on the treatment status and severity of HTN and diabetes. Instead, we matched the number of antihypertensive and antidiabetic drugs to balance the severity and treatment status of HTN and diabetes. Second, this administrative dataset lacks information on family history, physical activity, and alcohol drinking. This study included important variables, such as age, sex, obesity, smoking status, comorbidity, diabetes complications, and medications, and used propensity-score matching to balance study and control groups. However, unmeasured or unknown confounders may still affect our results. Finally, this population-based study mainly included the Chinese population; thus, the results cannot be generalized to other ethnicities. This study was a retrospective cohort study with some inevitable bias, and prospective randomized studies are needed to verify our results.

## Conclusions

Our study demonstrated that diabetes participants with subsequent HTN and HTN participants with subsequent diabetes showed significantly higher risks of CVD. In addition, the magnitudes of the point estimated CVD risk of HTN participants, and T2DM participants with subsequent HTN showed higher risk than T2DM participants without subsequent HTN. HTN seemed to have a greater impact on CVD risk. Participants should maintain healthy lifestyles to prevent the development of HTN and reduce the occurrence of future cardiovascular events.

## Data Availability

Data of this study are available from the National Health Insurance Research Database (NHIRD) published by Taiwan National Health Insurance (NHI) Administration. The data utilized in this study cannot be made available in the paper, the supplemental files, or in a public repository due to the ‘‘Personal Information Protection Act’’ executed by Taiwan government starting from 2012. Requests for data can be sent as a formal proposal to the NHIRD Office (https://dep.mohw.gov.tw/DOS/cp-2516-3591-113.html) or by email to stsung@mohw.gov.tw.

## Abbreviations

T2DM: Type 2 diabetes mellitus
HTN: Hypertension
CAD: Coronary artery disease
CVD: Cardiovascular disease
HbA1c: Hemoglobin A1C
AGE: Advanced glycation end-product
ROS: Reactive oxygen species
CKD: Chronic kidney disease
COPD: Chronic obstructive pulmonary disease
CCI: Charlson comorbidity index
DCSI: Diabetes complication severity index
